# Challenges at work and financial rewards to stimulate longer workforce participation

**DOI:** 10.1186/1478-4491-7-70

**Published:** 2009-08-11

**Authors:** Karin I Proper, Dorly JH Deeg, Allard J van der Beek

**Affiliations:** 1 Department of Public and Occupational Health and EMGO Institute for Health and Care Research, VU University Medical Centre, Amsterdam, The Netherlands; 2 Department of Psychiatry and EMGO Institute for Health and Care Research, VU University Medical Centre, Amsterdam, The Netherlands

## Abstract

**Background:**

Because of the demographic changes, appropriate measures are needed to prevent early exit from work and to encourage workers to prolong their working life. To date, few studies have been performed on the factors motivating continuing to work after the official age of retirement. In addition, most of those studies were based on quantitative data. The aims of this study were to examine, using both quantitative and qualitative data: (1) the reasons for voluntary early retirement; (2) the reasons for continuing working life after the official retirement age; and (3) the predictive value of the reasons mentioned.

**Methods:**

Quantitative data analyses were performed with a prospective cohort among persons aged 55 years and older. Moreover, qualitative data were derived from interviews with workers together with discussions from a workshop among occupational physicians and employers.

**Results:**

Results showed that the presence of challenging work was among the most important reasons for not taking early retirement. In addition, this motive appeared to positively predict working status after three years. The financial advantages of working and the maintenance of social contacts were the reasons reported most frequently for not taking full retirement, with the financial aspect being a reasonably good predictor for working status after three years. From the interviews and the workshop, five themes were identified as important motives to prolong working life: challenges at work, social contacts, reward and appreciation, health, and competencies and skills. Further, it was brought forward that each stakeholder can and should contribute to the maintenance of a healthy and motivated ageing workforce.

**Conclusion:**

Based on the findings, it was concluded that measures that promote challenges at work, together with financial stimuli, seem to be promising in order to prolong workforce participation.

## Background

One of the most notable changes in the working population is its ageing. The baby boom cohorts born after the Second World War (born between 1945 and 1965) are now middle-aged; the oldest of them have already started retiring. At the same time, lower birth rates in the past few decades imply that fewer young people will be entering the labour market [1]. These demographic changes are bringing about a shift in the ratio of workers to retirees that will lead to a relative shortage of active workers.

Of the major regions of the world, the process of population ageing is most advanced in Europe [2]. The median age of European Union (EU) citizens will increase between 2004 and 2050 from 39 to 49 years [3]. After 2010, the year that will mark the greatest number of members of the potential working population (i.e. those between 15 and 64 years), the population of working age will decline from 331 million to 268 million in 2050 [3]. In contrast, the proportion of people 65 years and older will increase.

These two demographic changes will result in an increase in the old-age dependency ratio (i.e. the number of people over 65 divided by the number of working-age people) from 25% today to about 53% in 2050 for the 25 EU countries [3]. At the same time, the share of older workers (i.e. those between 55 and 64 years) in the total potential workforce will logically increase. It is estimated that by the year 2025, between one in five and one in three workers will be an older worker [2].

It is clear that the demographic shift has serious economic and social implications, among others the financing of the social security systems, in that a shrinking number of economically active people (i.e. workers) will have to pay for the national pensions of an increasing number of retired persons. The ageing of the workforce also implies a change in the human resources (HR) strategies to manage age in the workplace. Thus, both government and private companies face the challenge of finding means to prolong the labour participation of (older) workers. This is especially true since, to date, many older people have left their jobs either voluntarily (i.e. because of early retirement) or involuntarily (i.e. because of work disability or unemployment) much earlier than the official age of retirement [4].

As in many countries, the social security system in The Netherlands used to offer the opportunity of retiring with a pension before the official retirement age of 65. This so-called early retirement pension (ERP) was implemented during a period of widespread unemployment, with the intention of providing better opportunities for the younger generation to find jobs. However, due to the population's ageing and its consequences, these early exits from work are no longer affordable from an economic point of view. Instead, appropriate measures are needed to prevent early exit from work and to encourage workers to prolong their working life.

During the last few years, measures discouraging early retirement have been implemented in many countries worldwide. For example, since 2006, ERP is no longer supported fiscally in the Netherlands, so that voluntary early exit from work has become more expensive. In other countries, raising the mandatory retirement age is one of the measures implemented. It may, however, be questioned whether such measures imposed by the government or the employer are effective. Commitment from the target group, i.e. the (ageing) workforce, is an important aspect for successful implementation.

To date, most of the research has focused on the determinants of early exit from work [5,6]. As far as the authors are aware, there are only limited data as to the motives of employees for prolonging working life. For example, a study of Lund and Borg [7] showed that very good self-rated health and high development possibilities were independent predictors for remaining at work among males. Among females, the same two predictors were found in addition to high decision authority, medium-level social support and absence of musculoskeletal problems in the knees [7].

In addition, some other recent studies showed that retirement decisions are influenced not only by the worker's health status, but also by income levels and pension rights [8,9]. Those aged 50 and over with poor health, high income or accumulated wealth and access to occupational pensions are more likely to retire at the normal retirement age or retire early [8,9]. Another study showed domestic and family considerations to be important influences of retirement behaviour [10]. In contrast, the evidence about the determinants of involuntary exit from work due to work disability shows occupational factors to be among the most important determinants [11,12].

However, the evidence as to the reasons for voluntary early exit from work is more scarce. From the few previous studies, it can be concluded that retirement decisions on a voluntary basis are multidimensional and not driven by any one single factor. In addition, the little available evidence as to the reasons for voluntary early retirement as well as for continuing working life is based mostly on quantitative data. There have been only a few attempts that involved qualitative data incorporating the worker's opinions about the factors that motivate them to prolong their work career after the official age of retirement.

For example, a semistructured interview study among persons who chose early retirement and those who did not, supported the quantitative finding that decisions about retirement are not made in a vacuum, but have to do with diverse types of possible routes into retirement [13]. These dealt with organisational restructuring, financial offers and opportunities for leisure and self-fulfilment that early retirement offers [13].

From a second qualitative study, it appeared that the way of conceiving work and retirement varied among persons from different socioeconomic backgrounds [14]. The conclusion was that various factors, including financial imperatives and HR practices, intersect at state pension age to shape people's routes into retirement and their options for continuing in work [14].

Finally, most of the previous studies have used cross-sectional data. Hence, the predictive value of the motives to retire early mentioned by those still working remains unclear.

Based on the limited literature on the determinants for prolonging working life, and the scarcity of qualitative data, the aims of the present study were: (1) to examine the reasons for voluntary early retirement; (2) to examine the motives for continuing working life after the official retirement age; and (3) to examine the predictive value of the reasons mentioned. A mixed-methods approach was applied, with quantitative and qualitative data.

## Methods

This article describes the results of three studies. The first study includes data analyses of a prospective study among persons aged 55 years and older. The second study is based on qualitative data from interviews with workers, while the third study includes a workshop among occupational physicians (OPs) and employers.

### Study 1. Quantitative study (LASA)

The aim of this quantitative study was to examine the reasons for voluntary early retirement (first study aim) as well as the reasons not to voluntarily retire early. Moreover, with the data of both baseline and follow-up (i.e. three years later), the predictive value of the motives stated at baseline was determined (third aim).

#### Study sample

The first study sample consisted of participants of the Longitudinal Aging Study Amsterdam (LASA). LASA is an ongoing, multidisciplinary, cohort study among persons aged 55 and over. It focuses primarily on the predictors and consequences of changes in older persons' physical, cognitive, emotional and social functioning.

The sampling and data collection procedures and the response rates were described in detail elsewhere [15,16]. In summary, LASA started with data collection in 1992–1993. A random sample of persons aged 55 years and over (birth years between 1908 and 1937), stratified by age, sex, urbanisation grade and expected five-year mortality was drawn from the population registers of 11 municipalities in three regions in The Netherlands. This procedure led to a representative sample of the Dutch older population, reflecting the national distribution of urbanisation and population density.

In 2002–2003, a second sample of men and women aged 55 to 64 years was drawn with the same sampling frame as the original cohort. The 2002–2003 sample is the sample for the current study, comprising 1002 participants aged 55–64 years (response rate 57%). In 2005–2006, a follow-up measurement of the second cohort took place (n = 908).

Written informed consent was obtained from all participants. The study was approved by the Medical Ethics Committee of the VU University Medical Centre.

#### Interviews

The interviews were held at the respondents' residences and were conducted by trained interviewers, who used laptop computers for data entry. The structured interview covered a wide range of topics related to physical and cognitive health and social and psychological functioning. For the purpose of the present study, the interview also included questions on reasons for considering early retirement.

The respondents were asked several questions as to their working status. Questions relevant to this study were: (1) Are you currently working in a paid job? (yes; no); (2) Are you currently (partially) work-disabled? (yes; no); (3) Have you already taken (partial) early retirement? (yes, completely; yes, in part; no); and (4) Would you consider taking (partial) early retirement if financially possible? (yes; no). Partial early retirement refers to working fewer hours in the main occupation.

**Figure 1:**
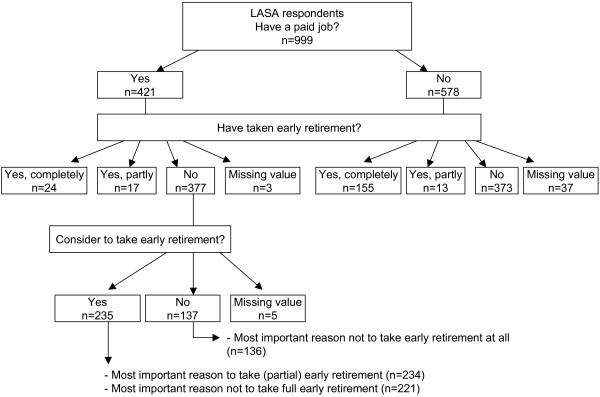
**Flow diagram of the LASA respondents regarding working and retirement status**.

To get insight into the reasons for voluntary early retirement as well as the reasons not to voluntarily retire early, respondents were asked their most important reason: (1) to take (partial) early retirement; (2) not to take full early retirement; (3) not to take early retirement at all; and (4) (among those who had already taken (partial) early retirement) to have taken (partial) early retirement. All four questions included branching questions that were asked to subgroups according to the working status and the consideration to take (partial) early retirement (Figure 1). The first two questions were asked of those with a paid job, who had not already taken early retirement, but who were considering taking early retirement, whereas the third question was asked of those with a paid job, who had not already taken full retirement and who were not considering taking early retirement. For each of these questions, the last of the five or seven answer categories included "another reason" than mentioned, leaving respondents space to fill in their own reason.

#### Analysis

For the purposes of this study, descriptive analyses were conducted. A frequency table was produced for each of the four questions (see above) indicating the percentage of each reason specified. The "other reason" category was analysed in more detail; answers that could be clustered were grouped. The predictive value of the reasons reported was determined by a frequency table of the working status (i.e. working or having retired) at follow-up per reason mentioned at baseline. Analyses were performed using SPSS software, version 14.0 for Windows.

Both Study 2 and Study 3 were performed to get insight into the motivating factors for continuing to work and the measures that can be taken to stimulate prolonging working life.

### Study 2. Interviews with workers

#### Study sample

Workers were recruited by the occupational health service (OHS) that participated in this study. By means of its customer database, the OHS approached 12 companies that differed in sector and size. Four companies agreed to participate. These four companies were from different sectors and included: (1) local government, (2) youth- and health care, (3) outdoor advertising and (4) an OHS, located in another city than the one involved in the recruitment in this study. The companies also varied in size (from approximately 60 to 700 workers), job characteristics and workers' educational level.

Within each company, the aim was to interview two workers individually and to hold one focus group interview with approximately five to 10 workers. Selection of the workers was done by a member of the HR staff or the head of the department, and was based on socioeconomic factors (e.g. sex, age, job position), availability and willingness to volunteer, to capture a broad range of characteristics. The workers were approached primarily face-to-face by their HR staff or supervisor to participate in the interviews. After agreement, the researcher arranged a specific date and time for the interviews.

#### Interviews

For the purposes of this study, semistructured interviews were held. Semistructured interviews define the area to be explored, at least initially, and allow the interviewer or the interviewee to diverge in order to pursue an idea in more detail [17]. This strategy encourages open answers, thereby eliciting new, additional information. During the interview, the interviewer tried to be interactive and to uncover factors that were not anticipated at the outset of the interview.

After briefly introducing the study and asking a few general questions, the interview guide posed the following questions: (1) "Are you willing to continue working until or after the age of 65?" (2) "What are factors that motivate you to continue working?" (3) "What measures can or should be taken to prolong your work career?"

All interviews took place face-to-face and were recorded on a digital voice recorder. The focus group interviews lasted approximately 50 minutes; the duration of the individual interviews varied from 24 to 42 minutes. During the interviews, the interviewer took field notes. The interviews were held in a meeting room at the company and were conducted by the principal researcher (KP).

#### Analysis

The interviews were fully transcribed by an assistant. Subsequently, content analysis was conducted by the principal researcher to analyse the transcripts. First, the transcripts were read and reread to become familiar with the text. Next, the text was marked with codes indicating the content of the response. The codes were then grouped together into key themes. In the Results section, interviewees' quotations that were considered representative for the theme are reported in order to illustrate the meaning of the themes.

### Study 3. Workshop with occupational physicians (OP) and employers

#### OPs and employers

To compare the views of the workers with the opinions of important stakeholders, a two-hour workshop among OPs and employers was held. The workshop was organised within a general course for OPs by their occupational health service (OHS). As the workshop fit well in the programme, it was decided to incorporate the workshop in the OHS's OP course. In total, 20 OPs participated in the course, including the workshop. In addition, five representatives (human resource management (HRM) staff) of the four participating companies joined during the workshop.

#### Workshop

The workshop started with a 30-minute presentation by the principal researcher about the study and the results of the interviews among the workers. Subsequently, four working groups were formed, each consisting of one or two representatives of each company and five OPs.

For one hour, each working group discussed two issues. First, they discussed the motivating factors mentioned by the workers. In their discussion, the OPs and employers were encouraged to add motivating factors. The second issue discussed in the working group concerned the measures to be taken by the employer or the OHS that might stimulate workers to prolong their participation in the workforce. Each working group was asked to write down its views, and one person within each group was asked to report on these in the plenary session. In the plenary session, per working group, the workshop leader (KP) wrote down all views of each working group on a flip chart and gathered the papers of each working group.

#### Analysis

After the workshop, the views reported on the flip chart and the working groups' papers were copied by the researcher in an electronic form on a computer. The workshop notes were coded according to the themes identified by the interviews with workers. Similar to the analysis of the interviews (Study 2), the text was marked with codes and then grouped together into themes.

## Results

### Study 1

Table 1 shows the working status of the study population at baseline. A small majority (57.9%) did not have a paid job (any longer) at the moment of baseline measurement, and about a quarter of the respondents (23%) were work-disabled. The large majority (78.2%) had not taken early retirement. Of those currently working (n = 421), almost two thirds (63.2%) reported they were considering taking (partial) early retirement (Table 1). Further, among those with a paid job, n = 377 were not yet partially retired early (Figure 1).

**Table 1:** Working status of the study sample (LASA cohort 2002–2003) at baseline

	**% (n)**
*Have a paid job*	n = 999

No	57.9% (578)

Yes	42.1% (421)

*Have a (partly) work disability*	n = 961^1^

No	77% (740)

Yes	23% (221)

*Have taken early retirement*	n = 960^1^

No	78.2% (751)

Yes, partly	3.1% (30)

Yes, completely	18.6% (179)

*Consider taking early retirement*	n = 372^2^

No	36.8% (137)

Yes	63.2% (235)

^1^Due to missing values, the number of respondents is not equal to 999.

^2^This question was asked of those currently working and not having taken early retirement.

Table 2 presents the frequencies of the workers' most important reasons not to take (full) early retirement. From this table, it can be concluded that the reasons for not taking early retirement at all are different from the reasons for not taking full early retirement. Having sufficient challenges at work appeared to be by far (59.6%) the most important reason for workers not to take early retirement, whereas the financial aspect (32.6%) and the social contacts (25.3%) were reported most frequently as the most important reasons not to take full early retirement (Table 2).

**Table 2:** Frequency of most important reason not to take (full) early retirement

	**Most important reason not to take early retirement**^1^	**Most important reason not to take full early retirement**^2^
Most important reason reported at baseline	% (n)	% (n)

Sufficient challenges at work	59.6 (81)	18.1 (40)

Maintain social contacts	17.6 (24)	25.3 (56)

Other pastime less pleasant	0.7 (1)	1.8 (4)

Financially more favourable	5.9 (8)	32.6 (72)

Other reason	16.2 (22)	22.2 (49)

	100% (136)	100% (221)

^1^This question was asked of those with a paid job, who had not taken early retirement, and who were not considering taking early retirement.

^2^This question was asked of those with a paid job, who had not taken early retirement, but who were considering taking early retirement.

Table 3 presents the predictive value of the reasons mentioned for work status at three-year follow-up. It appeared that the majority of those who reported challenges at work as the most important reason not to take (full) early retirement, were indeed still working three years later (84.4% and 66.7%) (Table 3). With respect to the financial advantages as the most important reason not to take full early retirement, it appeared that three years later, 68.3% were indeed still working or partly retired, but a quarter (24.2%) had taken full retirement. The maintenance of social contacts had less predictive value, since one third (35.4%) of those who reported social contacts as the most important reason not to take full retirement, had taken full retirement in the meantime.

**Table 3:** Working status at follow-up per reason not to take (full) early retirement as reported at baseline

	**Working status at follow-up per reason not to take early retirement**			**Working status at follow-up per reason not to take full early retirement**			
	working	partly/fully retired early	disabled	working	partly retired early	fully retired early	disabled
Reason reported at baseline	% (n)	% (n)	% (n)	% (n)	% (n)	% (n)	% (n)

Enough challenges at work	84.8 (56)	7.6 (5)	7.6 (5)	66.7 (20)	5.9 (2)	11.8 (4)	13.1 (4)

Maintain social contacts	80 (12)	0 (0)	20 (3)	32.5 (13)	10.4 (5)	35.4 (17)	17.5 (7)

Other pastime less pleasant	100 (1)	0 (0)	0 (0)	66.7 (2)	0 (0)	25 (1)	33.3 (1)

Financially more favourable	66.7 (4)	16.6 (1)	16.6 (1)	63.8 (37)	4.5 (3)	24.2 (16)	8.6 (5)

Other reason	84.6 (11)	7.7 (1)	7.7 (1)	46.3 (19)	8.9 (4)	33.3 (15)	9.8 (4)

Total	n = 84	n = 7	n = 10	n = 91	n = 14	n = 53	n = 21

Table 4 describes the most important reasons for taking early retirement among workers as well as among those who had retired early. Among the workers, the pleasure of spending more time on private concerns was by far the most important reason to take early retirement (59.4%). This reason was also reported most frequently by those who had already taken (partial) early retirement (27.3%) (Table 4). Further, among those who had taken early retirement, external factors, such as arrangements that made early retirement attractive and organisational changes, were also reported frequently as the most important reason to have taken early retirement. Health complaints as well as (physical or mental) workload were reported by a only small minority of the workers and retirees (<10%) (Table 4). Although the pleasure of spending more time on private pursuits was reported frequently as the most important reason to take early retirement, the majority (65.7%) were still working at follow-up (data not shown).

**Table 4:** Frequency table of most important reason to take (full or partial) early retirement

	**Workers**^1^	**Early retirees**^2^
Most important reason reported at baseline	% (n)	% (n)

Stress and pressure of work too high	9.8 (23)	6.3 (13)

Physically too heavy	6.8 (16)	5.4 (11)

Health complaints too limiting	6.8 (16)	6.3 (13)

Not motivated anymore	2.1 (5)	5.9 (12)

Nicer to spend more time on private life	59.4 (139)	27.3 (56)

Not possible anymore in the future	1.7 (4)	5.4 (11)

Having worked for many years^3^	2.1 (5)	-

Organisational changes in company^3^	2.6 (6)	9.8 (20)

Arrangements that made early retirement attractive^3^	-	14.6 (30)

Other reason	8.5 (20)	19.0 (39)

	100% (234)	100% (205)

^1^This group included those with a paid job, who had not taken early retirement, but who were considering taking early retirement.

^2^This group included those who have already taken (partial) early retirement.

^3^This category was formed after clustering the answers of "other".

### Study 2

#### Interviews with workers

Thirty workers were interviewed, either individually or in focus groups. With the exception of the local authority, within each company two interviews were held, each with one worker, as well as one focus group interview with five to eight workers. At the local authority, it was not possible to hold a focus group interview; instead, five workers were interviewed individually. Thus, a total of 11 individual interviews (six men, five women) and three focus group interviews were held among 19 workers (nine men, 10 women) aged 30 to 59 years.

Although the questions in Study 1 differ from those in the qualitative study (i.e. questions related to the reasons either to take or not to take early retirement versus the motivating factors to continue working), the results were rather similar. In line with the LASA results, where only about one third of those currently working were not considering taking early retirement, it appeared from the interviews that most workers were not willing to continue working after the age of 65 years. Although the majority of the interviewees indicated that they were still motivated to work, that they liked their job and that they (still) were healthy enough to perform their job, they did not intend to prolong their working life.

Furthermore, the major reasons (i.e. sufficient challenges at work, maintenance of social contacts and the financial aspect) reported by the LASA respondents for not taking (full) early retirement were also expressed by the interviewees as motivating factors to continue working. From the responses of the interviews, five key themes were identified: (1) challenges at work, (2) social contacts, (3) reward and appreciation, (4) health and (5) competencies and skills (Table 5). The themes include predominantly motivating factors, but also point to measures that can be taken to stimulate a sustained employability.

**Table 5:** Working status at follow-up per reason not to take (full) early retirement as reported at baseline

**Themes**	**Motivating factors**
Challenges at work	- Work climate is important - Being needed, feel oneself useful - Commitment to work and company - Work should be challenging and give satisfaction - Deliver a quality product

Social contacts	- Social contacts - Socially active

Reward and appreciation	- Financial compensation or reward at the sort term - Appreciation for the work done (by giving compliments)

Health	- Prevention of work strain (physically and mentally) - Healthy lifestyle - Optimal balance between work load and capacity

Competencies and skills	- Moving possibilities within company (horizontal and vertical) - Variation in tasks - Career support - Education and training - Coaching role for older worker - Retraining, occupational resettlement

#### Challenges at work

Most of the interviewees considered the content of their job of importance to continuing to work. They indicated that they liked their job, were motivated by their work and that they needed their work. With the exception of the workers who performed a physically heavy job, which included routine, it was frequently indicated that they perceived a feeling of satisfaction and motivation when they were being challenged.

By nature, I am rather lazy, but I am challenged by my work. Being at work, I become challenged intellectually; without work, there is no interesting life for me."

"So far, I am not ready to stop. I am motivated to work, to continue work, because the job is challenging enough."

#### Social contacts

Without exception, all workers interviewed appeared to set great store on the contact with colleagues and the associated work climate. One worker, for example, expressed this motive as: "Work is both intellectual and social food." Another worker reported: "It can be that your 'world will become so narrow' ... yes, the contact with colleagues and clients is very important."

Reward and appreciationMost of the interviewees highly valued appreciation from others for the work they did, and considered it as an important factor in continuing. This motive referred to both the financial aspect and reward expressed in words by the supervisor or colleagues. Although none of the interviewees indicated the financial reward as the most important reason to continue working, they agreed that "it definitely plays a role". One worker said: "Respect and appreciation, that's what I think is important."

As to the pat on the back (by the boss) as a motivating factor, they valued receiving a compliment from either the supervisor or colleagues. For example, one worker said:

"I absolutely think reward is essential in remaining motivated to perform the job. This can be through a bonus, but also by your colleagues who say to you how well you performed the task, or by having a dinner together, or something like that."

#### Health

In the company providing outdoor advertising, the interviewees performed heavy, physical jobs. These workers generally had a negative attitude about prolonged participation in the workforce. One reason for this negative attitude was associated with the total years of having worked when they reached the age of 65 years, since they had started working when quite young. Another reason for their negative attitude concerned the expectation that they would not be able to continue their (current) work, due to the heavy physical workload. Because of their workload, these workers suggested using tools that would reduce the physical work in order to be able to prolong participation in the workforce.

In the remaining three companies, physical workload was not the issue, in contrast to mental workload. Especially in the OHS, workers experienced (too) high work demands. To reduce or cope with work-related stress, some workers suggested implementing a relaxation programme or creating possibilities for relaxation, e.g. by means of a room where workers could rest, or through implementation of a yoga programme.

The promotion of a healthy lifestyle, including physical activity and diet, was also mentioned frequently as being an important factor for increasing the capacity and motivation to prolong a healthy working life. Although they generally agreed that a healthy lifestyle was the worker's own responsibility, they also agreed on the role of the employer in stimulating as well as facilitating such a lifestyle.

"I need to take care that I stay healthy; that's my own responsibility."

#### Competences and skills

Finally, the interviewees agreed on the value of education and training of (older) workers to be able to keep up with technological developments. They also reported that training or education was valuable and should be offered by the employer in order to grow (personally), to stimulate challenges at work and to avoid routine work.

"One needs to develop oneself; as soon as the job becomes a routine, it's not good, and one will not remain motivated."

It was further suggested to include the competences and personal development in the functioning discussions:

"In my opinion, the personal development should be included in the yearly functioning discussion."

There were no substantial differences in factors stated by younger and older workers. It appeared only that younger workers had difficulties in describing factors that would motivate them to prolong their working life, as "it is such a long way off".

### Study 3

#### Workshop with OPs and employers

The OPs and employers generally agreed with the workers' opinions expressed in the interviews. No additional factors were mentioned by them.

As to possible measures to be taken by the employer or the OHS to prolong workers' participation in the workforce, the working groups generally agreed with each other and reported more or less the same measures. From the notes, the following main themes were identified: (1) health promotion, (2) education and training and (3) financial stimuli.

#### Health promotion

Each working group independently reported factors that involved promoting the balance between workload and individual capacity, the latter receiving a notable amount of emphasis. The workshop participants not only referred to the promotion of physical activity and exercise, but also emphasised the role of a healthy diet, quitting smoking, a moderate consumption of alcohol and relaxation. Similar to the workers, they agreed on the responsibility of the worker, but also considered the role of the OP and the employer. One working group said, for example:

"It is of importance to stay fit and healthy; this is the responsibility of the worker. The employer, on the other hand, should give the good example. There should be attention for a healthy lifestyle within the organisation."

Another working group expressed its opinion about this issue as follows:

"The employer will do right if he implements a 'vitality policy' including physical activity, fitness, walking in lunchtime or walking during meetings. In most cases, the corporate culture needs to be changed in that it promotes health management with even more stringent measures when neglecting certain activities."

In addition to offering lifestyle programmes and providing information, they considered a periodic health screening to be a useful OHS tool, since the results of such a screening can give direct cause to providing (lifestyle) counselling. With respect to the other side of the balance, i.e. the workload, all agreed that this should be tuned to individual capacity.

#### Education and training

The working groups stated that work should be fun and offer sufficient challenges. This could, for example, be achieved by making plans about the work career and education needed and to be followed. Education and training should also be promoted, as it created variation in work, the latter being an important boost to taking pleasure in work. To achieve variation in work, some in the working groups suggested exchanging workers from different companies, or to give older workers a coaching or mentor task in orienting new colleagues.

To illustrate, one working group indicated:

"It is of crucial importance that one enjoys the job! This can be realised by several measures – among others, by giving older workers a coaching task in which they train young workers; the employer can also make agreements with the (older) worker about career planning."

#### Financial stimuli

Consistent with the interviews among the workers, attention was paid to the financial aspect. The OPs and employers agreed on the desirability of having both the employer and the government provide financial stimuli to workers who prolonged their working life. Moreover, they advocated maintaining the same net salary when demoting workers because of a (age-related) reduction in work ability.

## Discussion

The aim of this study was to examine the reasons for voluntary early retirement as well as for prolonging working life after the official retirement age. Insight into these motives is useful, among other reasons, as input to the HRM policy to retain healthy (older) workers who are able and willing to prolong their labour force participation. Despite the need to tailor the HRM policy to the needs and preferences of older workers [18], it should be kept in mind that older workers are a heterogeneous group in that differences exist in personal characteristics, needs and work ability between individual workers. This was confirmed by the OPs and employers in the present qualitative study, in that they stated that the workload should be tuned to individual capacity.

From the LASA analyses, it was shown that, of those currently working, about two thirds were considering taking early retirement. In view of the economic need to prevent early exit from work, this proportion is substantial. As mentioned before, it is important to encourage workers not to take early retirement, but to prolong working life instead. In order to achieve this, workers should be able as well as be motivated to continue working.

Indeed, the most important reason given by the LASA respondents currently working for not taking early retirement appeared to be the motivation to perform the job, i.e. the presence of challenge at work. This was supported by the interviews among the workers, where pleasure at work was mentioned frequently as a motive to prolong working life. Based on the LASA follow-up data, it appeared that the presence of sufficient challenges at work positively predicted the working status three years later. That is, the majority of workers who found their work challenging, and indicated this was an important reason not to take early retirement, actually remained in the workforce.

Another notable result of this study concerned the fact that the reasons for not taking full early retirement differed from the reasons not to take early retirement (at all). Based on the LASA analyses, the main reasons for the former were the financial aspect of working and the maintenance of social contacts. The social aspects, but particularly the financial advantages of working, thus seemed to play an important role in the decision to either fully retire or to cut down work gradually by partial early retirement.

Again, these results were confirmed by the qualitative study. The large majority of the workers interviewed said the social contacts with colleagues and others at work were of great importance to work motivation. However, based on the LASA follow-up data, it appeared that the maintenance of social contacts had a weak predictive value for the working status three years later. A substantial part of those who had reported social contacts being the most important reason not to take full retirement, did take full retirement in the meantime.

As to the financial aspect, both the workers interviewed and the OPs and employers agreed on its significance. In addition, the financial advantage of not taking retirement appeared to reasonably predict the working status three years later.

Based on these findings, including the desire of the majority of workers to take early retirement, it seems sensible to aim for a gradual exit from work through a period of working fewer hours in order to prevent full early retirement. In this way, the workers benefit financially and moreover can spend some more time on private pursuits, this being the most important reason to take early retirement, as shown by the LASA analyses. This was confirmed by a recent study by Cebulla et al. [19], which stated that there is broad consensus that older workers should be given the opportunity to retire gradually.

Another study found control over the retirement decision to be an important factor in retirement well-being, which persisted three years after retirement [20]. They also found that gradual retirement was followed by a positive change in health. Gradual retirement was linked to an improvement in health. According to the authors of that study, those findings suggest that HR practices that promote employees' control of their retirement decisions will enhance well-being in later life and facilitate prolonged workforce participation [20].

As mentioned in the introduction, not much literature has been published as to the reasons for voluntary early exit from work or for an enduring working life. However, the few previous studies performed in different countries support our results.

For example, Armstrong-Stassen (2006) investigated the importance of six HRM strategies relevant in the retention of older workers [21]. These six factors were: (1) flexible working options, (2) training and development, (3) job design (e.g. reduce workload), (4) recognition and respect, (5) performance evaluation and (6) compensation (e.g. financial, incentives). Although different terms have been used in Table 5, most of the five strategies, if not all, are reflected.

From Armstrong-Stassen's study, it appeared that both retired and employed men rated three HRM practices the same as to their influence on the decision to remain in the workforce: (1) providing challenging and meaningful job tasks; (2) recognising experience, knowledge and skills; and (3) showing appreciation for doing a good job [21]. Further, data from a representative sample of the household population aged 50 and over in England, the English Longitudinal Study of Ageing (ELSA), confirm that influences on retirement are multidimensional, with economic incentives being an important, if not the most important, determinant for continuing working life [22].

Two other factors of influence appeared to be health and social issues. As to the financial aspect as a determinant of retirement behaviour, the literature is consistent [23]. Using ELSA data, Banks et al. (2007) found that both pension accrual and pension wealth are important determinants of the retirement behaviour of men aged 50 to 59. This was also valid among women of the same age, although it was somewhat weaker [24]. It further appeared that there was a U-shaped pattern of being in paid work by quintile of the wealth distribution, with those at the bottom and the top of the wealth distribution being less likely to be in paid work than those in the middle of the wealth distribution, but for different stated reasons: those with relatively low levels of wealth were most likely to stop working due to ill health [24]. Thus, ELSA showed that financial need may act as an incentive to continue working life, but that this varies with wealth, income and education levels [25].

### Strengths and limitations of the study

As mentioned before, the few previous studies regarding the reasons for early retirement and prolonging working life have mostly used quantitative research methods. One of the strengths of the present study is that it is based on mixed methods, with both quantitative and qualitative data. Such a triangulation method is useful as it approaches the same phenomenon by using different methods, each having its own value. Qualitative research techniques add vast amounts of relevant data [26].

There were, however, some limitations in our qualitative study. Although the transcripts were made by an assistant, the interviews were performed by one researcher, as were the analyses of the responses into codes and themes. Further, despite our not believing that it introduces bias, we did not use a computer software programme in which each item is compared with the rest of the data to establish analytical categories [26]. Instead, the interviewer herself read and reread the recordings, identified the codes and grouped the codes together into key themes. The analyses were performed manually, which was feasible and acceptable, instead of by means of a software package specifically designed for qualitative data management. Although the latter would have made the process easier, we assumed the chance to be small that the manual process would have yielded substantially different clusters. However, as is the case in all qualitative studies, the categorisation of the themes depended on the human factor (i.e. the researcher), and might well have resulted in different clusters if the process had been performed by another researcher.

Another strength of this study is that the quantitative study involved a longitudinal study among a representative sample of older adults in The Netherlands. The LASA follow-up data were useful to provide information about the predictive value of the motives reported at baseline to take (or not) early retirement. Overall, results indicated that the motives reported did not have a high predictive value. However, considering the small number of follow-up data used, prudence is needed for the conclusions as to the predictive value of the reasons mentioned. In this respect, it is also worthwhile to mention that the present study can be considered as a pilot study in that it is based on data of Dutch workers only. As the topic of ageing workers is of worldwide interest, a future study involving diverse countries with diverse policies to encourage a sustained participation in the workforce would be valuable.

Finally, although the involvement of different stakeholders in the qualitative study added value to the study in that extra input could be generated instead of involving only one target group, the number of the representatives of companies was low. Nevertheless, the inclusion of the four companies, which included different in type of workers and work activities, yielded useful information about the organisation of the (HRM) policy with respect to the promotion of an enduring participation in the workforce of older workers.

## Conclusion

Taken together, findings suggest that each stakeholder (i.e. the worker, the employer, the OHS and the government) should contribute to the maintenance of a healthy and motivated ageing workforce. In doing so, one should take into account the differences in strategies for different groups, such as different socioeconomic status groups or those at different ages. Due to the small size of the study, and especially the qualitative study, the present study could not draw such conclusions for different subgroups. So, further research examining the motivating factors to prolong working life among different groups is recommended.

In addition to their ability to work, workers' motivation is considered to be crucial in sustaining participation in the workforce. Workers who lack pleasure at work and are not motivated to prolong their working life, probably confirm the negative image of some employers about older workers, i.e. lower productivity and "waiting out one's time". There are still some employers for whom the negative effects of ageing prevail over the value of older workers, in that older workers are less productive or resist change or innovations. In order to oppose this view, the employer can and should implement simple measures, such as offering education and expressing appreciation to the personnel. Finally, measures that promote challenges at work, together with financial stimuli, seem to be promising in keeping older workers in the workforce.

## Competing interests

The authors declare that they have no competing interests.

## Authors' contributions

All three authors made a substantial contribution in the design of this specific study. DD was (and still is) involved and responsible for the design and data collection of the quantitative study (Study 1). KP and AvdB were involved in the design and the acquisition of the qualitative data (Study 2). KP performed the statistical analyses. All three authors read and approved the final manuscript for submission to this journal.
